# The Potential of Seaweeds as a Source of Functional Ingredients of Prebiotic and Antioxidant Value

**DOI:** 10.3390/antiox8090406

**Published:** 2019-09-17

**Authors:** Andrea Gomez-Zavaglia, Miguel A. Prieto Lage, Cecilia Jimenez-Lopez, Juan C. Mejuto, Jesus Simal-Gandara

**Affiliations:** 1 Center for Research and Development in Food Cryotechnology (CIDCA), CCT-CONICET La Plata, Calle 47 y 116, La Plata, Buenos Aires 1900, Argentina; 2 Nutrition and Bromatology Group, Department of Analytical and Food Chemistry, Faculty of Science, University of Vigo – Ourense Campus, E32004 Ourense, Spain; 3 Department of Physical Chemistry, Faculty of Science, University of Vigo – Ourense Campus, E32004 Ourense, Spain

**Keywords:** seaweeds, macroalgae, invasive species, prebiotics, antioxidants, functional foods

## Abstract

Two thirds of the world is covered by oceans, whose upper layer is inhabited by algae. This means that there is a large extension to obtain these photoautotrophic organisms. Algae have undergone a boom in recent years, with consequent discoveries and advances in this field. Algae are not only of high ecological value but also of great economic importance. Possible applications of algae are very diverse and include anti-biofilm activity, production of biofuels, bioremediation, as fertilizer, as fish feed, as food or food ingredients, in pharmacology (since they show antioxidant or contraceptive activities), in cosmeceutical formulation, and in such other applications as filters or for obtaining minerals. In this context, algae as food can be of help to maintain or even improve human health, and there is a growing interest in new products called functional foods, which can promote such a healthy state. Therefore, in this search, one of the main areas of research is the extraction and characterization of new natural ingredients with biological activity (e.g., prebiotic and antioxidant) that can contribute to consumers’ well-being. The present review shows the results of a bibliographic survey on the chemical composition of macroalgae, together with a critical discussion about their potential as natural sources of new functional ingredients.

## 1. Macroalgae Classification

We live on a planet of which ~72% of the surface is water. Since all the necessary elements for life are found in seawater, every form of life emerged from that immense original matrix. These elements and many others, since all the elements of the periodic table are in the sea, have the advantage of being present in quantities that are generally stable and constant along the marine surface, contrary to what happens on earth [1]. However, traces of our marine origins can also be found on earth, since there are similarities between the composition and some properties of the sea and those of biological fluids [2,3,4].

The oceans contain and give life to approximately 500,000 species, which means that almost three quarters of all known species inhabit seawater. Among them are algae which, although the vast majority inhabit salt water, can also survive in freshwater. These are very peculiar living beings, to whom the development of life on our planet is due, since the algae were pioneers of photosynthesis, thanks to the evolution of chlorophyll function 3200 million years ago [1,5]. Photosynthesis probably began in some blue-green prokaryotic microorganisms that were formerly considered algae, and that currently belong to the *Phylum Cyanobacteria*, which is included in the Monera Kingdom [6].

More than 30,000 species of algae have been described, and their scientific study is called phycology. According to the current definition of algae, the blue-green variety is not considered algae, as they are prokaryotic organisms, and only eukaryotic organisms belong to this category (either unicellular, such as microalgae phytoplankton, or multicellular, such as macroalgae) [3]. The taxonomic classification of algae is complex due to the number of existing varieties and the many applicable classification criteria. They are a polyphyletic group, which means that they belong to different kin groups. Therefore, the classification is not well defined, and may vary according to the authors, but they are currently included in the Protista kingdom, with some exceptions of macroalgae belonging to in the Plantae kingdom [7,8].

The microalgae, Protista microorganisms that are classed as phytoplankton, are important in nature because they represent the first trophic level in the food chain, serving as nutrients to thousands of marine species. They are also essential in chlorophyll function, since they are primary producers, being responsible for 30%–50% of the oxygen contained in the atmosphere [4].

Macroalgae are also a very varied group, with sizes ranging from a few centimeters to specimens that can reach 100 m in length. Approximately 15,000 species of this group have been described [9]. They are also autotrophic and photosynthetic beings, so their habitat is limited to a certain depth, which is usually a maximum of 60 m, always within the intertidal zone, and its growth is usually vertical, looking for sunlight [10,11]. The differences between them and terrestrial plants are that they do not present conductive tissues, but rather they adsorb nutrients throughout their whole surface. They also lack roots, though some present rhizoids or basal discs that allow them to adhere to rocks as a method of restraint, but not to nourish themselves. They form large underwater meadows and are generators of ecosystems in which many different species of bacteria, corals, mollusks, fish, and other marine creatures accumulate and coexist [7,12,13,14].

Much of the literature agrees that macroalgae can be divided into 3 large groups: The Chlorophytas, commonly known as green algae, the Rhodophytas or red algae, both included in the Plantae kingdom; and the Ochrophytas, mostly classified in the Phaeophyceae class. These are also called brown seaweed and belong to the Protista kingdom, as well as the microalgae kingdom. The classification of this algae macrogroup was made taking into account the pigment that composes it, and through which it manages to perform photosynthesis to carry out autotrophic feeding [13]. There are authors that include some green macroalgae in another differentiated group and at a taxonomic level equivalent to Chlorophytas, which are responsible for life expanding beyond the oceans, and are a precursor of terrestrial plants [15]. Table 1 summarizes the phylogeny of these four large groups of macroalgae. The mentioned differences between algae pigments are collected in Table 2.

## 2. The Potential of Invasive Seaweeds

Due to increasing globalization as well as climate change, the arrival of invasive algae to coastal areas of different regions is becoming more common. However, these are not the only causes of the increased proliferation of these non-indigenous marine species. Other causes, such as those related to the marine industry (aquaculture, fisheries, and marine tourism) must also be considered [18,19,20]. That is why marine biodiversity is seriously threatened by macroalgae invasions. In fact, macroalgae represent between 10% and 40% of the total number of species introduced into the marine ecosystem [21].

These algae not only cause an environmental problem by displacing other native species, causing a loss of biodiversity, damage in the structure and function of the native ecosystem, and a homogenization of the landscape, but also cause losses in the fishing, recreational sector, and many other industrial sectors related with aquatic environment. However, this abundance can also present opportunities. It is therefore of great interest to find possible applications to give added value to these algae. Furthermore, most of the foods humans eat in the modern world come from a small number of domestic animals and plants widely raised and cultivated, most of them having been introduced voluntarily by humans. One of these applications could be the obtainment of compounds of natural origin.

Because invasive algae pose a serious threat in coastal areas, the interest in developing protocols for the control of these exogenous species is booming, although in parallel, researchers are also trying to develop strategies that allow them to be used as a natural source of secondary metabolites. One of the measures proposed is the eradication of the species, which is a promising solution for areas declared as protected, such as Marine Protected Areas. In this sense, the enormous production of biomass by this type of algae can become a benefit, since it offers the possibility of recycling or reuse at the time of eradication. In any case, it should be taken into account that the impact-control studies do not always allow us to reach an accurate conclusion about the impact of a certain invasive algae, since these studies are designed mainly with the intention of evaluating the anthropogenic impacts, when a control-impact evaluation before and after the invasion would be more accurate [22].

The species of marine algae that are in the top five of “potentially invasive”, which means that they meet certain characteristics (relating to the mechanisms of reproduction, growth and defense, resistance to pollution, among others), as well as having a high ecological impact, are, from least to greatest: *Grateloupia turuturu* (as *Grateloupia doryphora*), *Asparagopsis armata, Undaria pinnatifida, Caulerpa taxifolia,* and *Codium fragile* subsp. *tomentosoides* [23]. Some authors [24] classify the factors that favor such invasive attacks of macroalgae in two groups: The abiotic ones, such as salinity and waves, and the biotic ones, such as those related to the competitive abilities of the species. Other authors [25] proposed the Tilman’s R* rule, where R refers to the resources available in an area and R* to the balance of available resources, from which one can predict where the invasion is favored, since if the R* of the endogenous species is greater than that of the exogenous species, the invasion is more likely to occur, and may occur in two ways: The invader needs fewer resources than the resident, or the range of acquisition of resources of the invader is greater than that of the endogenous one. On the other hand, there is a study [24] that discusses about the enemy release hypothesis (ERH), based on the fact that the invader has escaped from its habitat due to the presence of enemies and/or herbivores. This can also be the explanation of why invasive algae tend to have greater resistance to herbivores than native ones. For example, *Fucus evanescens*, presents higher amounts of phlorotannins, compounds known to cause animosity to herbivores [24], than the native species [26].

*Laminaria* sp. (brown algae) show a great adaptability and relocation thanks to their gametophytes, formed by filamentous tufts of approximately 1 or 2 cm. These reproductive structures can be considered as “seed stocks”, so their presence is extremely important when colonizing a certain area, as does, for example, the species *Undaria* sp. [27]. Regarding the interactions between the invasive species themselves, information is scarce, so it is an area still to be explored that concerns the entire globe, since invasive algae are not governed by national borders.

Many of these algae contain bioactive compounds that could be contemplated for a wide range of commercial applications like nutrition and pharmaceutical ones. In this regard, invasive algae present great advantages because, according to some authors [20], invasive algae present fast growth rates and biomass accumulation, high levels of repellency against herbivores, and often low levels of epiphytism. Moreover, according to these authors, the low cost of algae farming, along with good economic results and the high demand for products obtained from algae, has led to the intentional introduction of potentially invasive algae in overcoat low-wages countries.

## 3. Algae Aquaculture

Aquaculture is the science of cultivating aquatic animals, plants, and related organisms like fish, shellfish, seaweed, and microalgae, for human use and consumption, and would be a fast-growing industry. Nowadays, many authors are focused on performing Life Cycle Assessment (LCA) of seafood production to provide new insights into its environmental impacts and therefore to improve environmental sustainability of the aquaculture production systems [28]. Although the technologies related to aquaculture of algae have undergone tremendous development in the last 70 years, especially in Asia, but also in America and Europe, there is still much to improve regarding their science and the social acceptance that entails. One of the main challenges is the development of strains that are thermo-resistant, of rapid growth, with high production of compounds of interest, resistance to morbidity, and antifouling capacity, as well as the development of efficient and economical hatcheries, capable of withstanding storms in the open sea [29]. One fact to keep in mind is that algae aquaculture offers advantages to ecosystems, since it improves the conditions of coastal waters, favoring other species and the environment. Interestingly, although more than 10,000 species of algae are known, aquaculture of algae is mostly made (more than 81% of production) using very few of them [30]: The brown algae, *Saccharina japonica* and *Undaria pinnatifida*, and the red algae *Porphyra sp.*, *Kappaphycus alvarezii* and *Eucheuma striatum* (carrageenophytes), and *Gracilaria/Gracilariopsis* sp. (agarophytes).

One of the priorities when developing aquaculture is to be sustainable, that is, to ensure the minimum possible adverse effects for the environment. One way to achieve this is by developing improved methods for waste treatment. Integrated Multi-Trophic Aquaculture (IMTA) combines the aquaculture of food (for example, of fish) with that of extraction (for example, algae) to create a more balanced ecosystem [31]. However, caution should be exercised, since coastal waters containing high amounts of nutrients may favor the emergence of potentially harmful, invasive, or opportunistic algae, which may have negative consequences for the coastal zone [32,33,34]. There is another similar concept that excludes the feeding concept, called nutrient bioextraction, which can be applied even to urbanized estuaries, where excess nutrients are currently a problem. In both aquaculture systems described, algae can be used as a solution to eliminate inorganic nutrients (phosphorus compounds, nitrogen, carbon dioxide, and other compounds used for their metabolism), thus decreasing the negative impacts on the environment [35,36,37,38]. In this way, while algae are cultivated, the levels of nutrients in the water are reduced, so its acceptance is much greater by users and those who are positioned against aquaculture, since the presence of seaweed provides advantages in the aquaculture system, such as minimal environmental adverse effects and reduction of costs, due to the utilization of wastes to feed other levels [31].

## 4. General Current Seaweed Industrial Applications

General current seaweed industrial applications are important because these days consumers look for products with a natural origin. This is a new use of algae, which is in high demand. A brief and general bibliographic review allowed us to define the main biological activities (antioxidant, antibacterial, anti-inflammatory, antifungal/antiprotozoal, antiviral, cytotoxicity/antiproliferative, adipogenesis, MAA/UV protection, matrix metalloproteinases and blood fluidity) of algae (summarized next in Table 3).

### 4.1. Anti-Biofilm Applications

Anti-biofilm activity has been investigated with models based on Gram-positive and -negative bacteria. This is important because biofilms may cause diverse diseases. Several studies concerning anti-biofilm activity can be used as practical examples. For instance, there is a research about how seaweeds can attack *Salmonella enterica* biofilms. That is important because this organism is one of the most prominent causes of bacterial food-borne diseases. In that study, it was observed that brominated furanones obtained by extraction from the red algae *Delisea pulchra,* interfere in this biofilm formation, but more studies are needed to determine a long term efficiency [41]. Another work [40] reported that *Sesbania grandiflora* extract has anti-biofilm and anti-bacterial activity against *Staphylococcus aureus*. Antimicrobial, anti-biofilm, and antifouling properties of sulfated polysaccharides obtained from marine macroalgae were also studied using dental plaque bacteria as model [39].

### 4.2. Biofuel and Bioremediation Applications

Algae can also be used in the production of biofuels. However, for making the process of obtaining biofuel feasible economically, algae should present a co-production of some other components that could be used biochemically and that have a proven commercial value [42]. This approach would also decrease the cost of algae treatments and should mitigate the eutrophication of lakes. However, using algae as a fuel has several advantages. One of them is that a single algal biomass can be used to produce several different kinds of renewable biofuels depending on the treatment applied (anaerobic digestion and biodiesel derived from algal oil, among others) [32].

Regarding bioremediation, the accumulation of high amounts of organic and inorganic matter is a risk factor for many ecosystems, and therefore, for human health, that is why new solutions are sought to remedy this accumulation. Algae can be one remedy at the aquatic ecosystem level, since they participate actively in the control and biomonitoring of organic pollutants [48].

Algae are a group of great interest for this purpose, since they are ideal for the bioremediation of wastewater thanks to their culture being very economical and easy to achieve on a large scale, since they are capable of capturing a high percentage of metal ions. Furthermore, according to the large amount of biomass that is produced by algae growing in wastewater, a dual-purpose crop could be considered to bioremediate wastewater, and to obtain a production system for other substances of interest, such as compounds with bioactivities [47].

Another study [46] reported that certain algae act as “hyper-accumulators” and “hyper-adsorbents” with a high selectivity for different elements. They also contribute to an alkaline environment, leading heavy metals to precipitate during treatments.

However, in all cases it is necessary to control some parameters such as temperature, pH, nutrients, and availability, among others, to reach the optimum conditions under which algae show the best absorption, removal, and biodegradation of different pollutants [46,47,48].

### 4.3. Fish Feed Applications

Most of the fish feed used in aquaculture is made from other fish meat. This has an enormous disadvantage as fishes have to be fed twice and that costs a lot. Moreover, the world’s fish stocks are decreasing, and aquaculture is increasing, which makes that system unsustainable.

As for the nutritional aspects of microalgae, they are strongly dependent on culture conditions as well as on factors such as culture phase, temperature, and availability of nutrients [71].

There are evidences of good nutritional properties of algae biomass as a source of micronutrients or as a bulk feedstuff. Moreover, they have a positive effect on the physiological state of the larvae due to, for example, the diversification of bacterial flora [72].

The use of algae as a bulk feedstuff or as a supplement depends on the biomass availability, as well as its composition and cost [73]. However, their use should be limited to a certain concentration due to the amount of toxic metals they may have, such as arsenic, which is one of the main limiting factors.

The consumption of microalgae is carried out directly, in the case of mollusks and crustaceans, and indirectly in urine from previous ingestion by zooplankton species [71].

Algae can be used for animal feed as well. They are used among others in ruminants’ nutrition.

### 4.4. General Food Applications

The main use of algae is the one with direct food applications (“seaweed as a vegetable”). This use represents the main world market for algae. This is mainly due to the great consumption that exists in Asian countries, where they are traditional products of high consumption. The main seaweeds used as human food are [74]: Nori or purple laver (*Porphyra spp*.), aonori or green laver (*Monostroma* spp. and *Enteromorpha spp*.), kombu (*Laminaria japonica*), wakame, (*Undaria pinnatifida*), Hiziki (*Hizikia fusiforme*), mozuku (*Cladosiphon okamuranus*), sea grapes or green caviar (*Caulerpa lentillifera*), dulse (*Palmaria palmata*), Irish moss (*Chondrus crispus*), winged kelp (*Alaria esculenta*), ogo (*Gracilaria spp*.), and *Callophyllis variegate.* Algae products are consumed as food in different ways: Dried, in sushi, in soups, in salads, in tea, in mustard, in pasta, in breads, and many other similar food products.

Algae also contain some compounds that can be used as food ingredients. For example, phycocolloids are a group of natural polymers constituted by polysaccharides with the ability to form gels (hydrocolloid) derived from macroalgae. They are used in almost all industries (food, drugs, paintings, cosmetics, etc.) due to their physical–chemical characteristics. These are the second-most common use of algae. The majority of obtained hydrocolloids are alginate, carrageenan, and agar. According to a previous study [74], several red and brown algae are used in the production of three hydrocolloids: Agar, alginate, and carrageenan. These compounds are water-soluble carbohydrates mainly used to increase aqueous solutions viscosity, to produce different types of gels, to form water-soluble films, and to stabilize some products. Other functional ingredients present in algae are carotenoids (used as food colorants, feed supplements, and nutraceuticals), lipids, proteins, polysaccharides, and phenolic compounds [32].

### 4.5. Pharmacology and/or Medical Applications

Algae produce bioactive compounds with rich pharmacological potential. They generate these compounds as a response to environmental conditions or characteristics (competition for space, maintenance of unfolded surfaces, repulsion of predators, etc.) [75]. In recent years, seaweeds have been recognized as producers of an enormous range of biologically active compounds, but the bioactivity of the same species could vary depending on the geographical zone due to environmental and seasonal parameters [75].

#### 4.5.1. Contraceptive Activity Applications

Different studies [49,50] with extracts from red algae were carried out. These extracts were administrated to female rats in the first seven days of their pregnancies. As result, it was demonstrated that these extracts have post-coital contraceptive protection in the rats without showing any relevant adverse effect.

#### 4.5.2. Antibiotic, Antiviral, and Antifungal Activity

Algae’s antibiotic activity was demonstrated for several authors through tests of the extracts obtained from algae against Gram-positive and -negative bacteria. Finding these new sources of antibiotics is interesting because microorganisms are getting more resistant to drugs, so providing new drugs is of utmost importance nowadays. That is why novel natural antimicrobial compounds with high potential, good availability, less toxicity, and fewer adverse effects are required [56].

According to a recent article [55], isolated chemical compounds from marine seaweed have been shown to owe bioactivities such as antimicrobial, antioxidant, and anti-inflammatory properties, as well as anticoagulant and apoptotic effects. Viral diseases are still one of the main causes of death in the world. Treatments sometimes fail because of the side effects of infectious diseases or because of drug resistance. Therefore, the study of algae compounds with antiviral activity is of great interest. According to other authors [58,76], algae from the three large groups (green, brown, and red algae) produce interesting polysaccharides that show antibacterial activity against some pathogen bacteria such as *Aeromonas salmonicida* or *Pseudomonas aeruginosa*, both being of importance because of their high pathogenicity, resistance, and incidence in humans. Specifically, some red algae produce sulfated polysaccharides showing antiviral capacity against viruses responsible for different infectious diseases. As an example, red algae *Gracilaria gracilis* extracts have shown antimicrobial activity against *Bacillus subtilis*, an extreme conditions resistant bacteria [77]. One of the main areas of interest is to find new treatments towards herpes simplex virus type 1 (HSV-1), as it is an endemic disease, the virus is developing resistance, and other available drugs have side effects [57].

#### 4.5.3. Anticancer Activity

Actually, cancer is one of the principal causes of death around the world, thus, finding new treatments for this disease is a big challenge. Some studies have linked the anticancer capacity of a certain algae with the content of compounds with antioxidant properties.

Most studies of anticancer properties in algae have been performed on microalga extracts or fractions obtained using low-resolution methods (liquid–liquid extraction, solid–liquid extraction) [59].

Extracts from algae *Bryopsis* sp. contain some compounds (depsipeptides kahalalide A and F) whose future seems to lie with the development of kahalalide F for treatment of lung cancer, tumors, and AIDS. This is due to the fact that kahalalide F has anticancer properties, namely the control of tumors causing colon, lung and prostate cancer [58]. It happens the same with chondriamide A, a compound obtained from *Chondria atropurpurea* that presents antiproliferative activity towards human nasopharyngeal and colorectal cancer cells [60].

Other compounds present in algae, such as several polysaccharides with sulfur groups—e.g., fucoids—have also shown cytotoxic properties [78]. Another example are terpenes. A study carried out using *Chlorella sorokiniana* and *Chaetoceros calcitrans* extracts concluded than they present interesting activities compared to commercially available marine anti-cancer drugs [59]. *L. papillosa* algae was also studied and the conclusion was that it contains some bioactive compounds that could serve as a promising potential antioxidant, antimicrobial, and anticancer agents [56].

#### 4.5.4. Anticoagulant Activity

Phlorotannins and sulfated polysaccharides such as fucoidans in brown algae, carrageenans in red algae, and ulvans in green algae have been recognized as potential anticoagulant agents [61,62,63,64]. For example, in a study carried out by [79], they tested the effect of some polysaccharides rich extracts obtained from green algae *Ulva fasciata* and red algae *Agardhiella subulata* in the thromboplastin time and prothrombin time, resulting that both algal extracts showed that prolong those mentioned times during the coagulation cascade, avoiding blood coagulation. However, the anticoagulant activity has been mainly investigated in vitro or in mouse. Hence, and due to the important value of that property, more studies are needed [62].

#### 4.5.5. Anti-Inflammatory Activity

Several studies have demonstrated that some extracts and compounds from a wide variety of algae have potential anti-inflammatory compounds. For example, [66] studied the anti-inflamatory capacity of methanolic extracts obtained from red seaweed *Dichotomaria obtusata*. Other authors [65] reported the enhanced anti-inflamatory ability of brown kelp *Laminaria japonica* by fermentation with *Bacillus subtilis*. Likewise, [67] reported anti-inflammatory properties of several marine algae collected from the Mannar coast gulf (Tuticorin, South India).

#### 4.5.6. Antioxidant Activity

An excessive amount of reactive oxygen species may result in lipid peroxidation, which change the structure of body biomolecules, causing cellular disorders, premature aging, mutations or cell death. Different researches have demonstrated seaweed antioxidant capacity in vitro, attributed to the presence of new antioxidant compounds like carotenoids, certain polysaccharides, and polyphenols, which show scavenger activity, being able to neutralize those reactive oxygen species through their own oxidation, since their affinity to those oxidative compounds is very high [56,68,80,81,82,83,84,85].

As a curiosity that strengthens the utilization of algae as antioxidant tools, a study carried out by [86] in which fishes were fed with red algae *Gracilaria gracilis* powder, demonstrates that this nutritional input favors genetic expression of some antioxidant enzymes, like superoxide dismutase and catalase, what it also improves the state of the body’s immune system.

### 4.6. General Cosmeceuticals Applications

Another possible use is to obtain compounds with cosmeceutical bioactivity, that is, compounds to be used as ingredients for skin care products. Many of the invasive species have been demonstrated to include these compounds. However, metabolite amounts often vary according to geographical and seasonal conditions, so environmental variability has to be taken into consideration [87]. For that type of product, brown and red marine seaweeds are the most used species. Extracts rich in potential cosmeceutical ingredients, such as phlorotannins, polysaccharides, carotenoids, fatty acids, as well as bioactive proteins, vitamins, and minerals can be obtained from seaweed [52]. These compounds are incorporated into cosmetics to optimize their properties; their capacity to stabilize and preserve products and the bioactive activities of the compounds found (antioxidant, photo-protection, anti-wrinkling, anti-cellulite, moisturizing, and whitening) [88,89]. In vitro studies also demonstrated that these compounds are UV protective, or have an inhibitory effect on melanogenesis [51].

### 4.7. Other Applications

Traditionally seaweeds have also been collected and used by coastal communities as fertilizers. They are appropriate for that use since they have the characteristic of enriching the soil with N, P, and K, favoring agriculture, and they have the ability to promote seed germination, increase frost resistance, and improve resistance to fungal and insect pests [32,43,53]. The main species that are used for agricultural applications are *Ascophyllum nodosum*, *Ecklonia maxima*, *Laminaria sp., Lessonia* species, and *Macrocytis pyriphea* [74]. The most common extractive modality to obtain these interesting compounds is alkaline extraction for which potash is added and heat is applied [90].

Another interesting property of algae is that they are able to take many minerals from the sea, with no substantial differences in the quantity among different types of algae. The most important macroelements they contain are sodium, calcium, magnesium, potassium, chloride, sulfur, and phosphorus. Regarding oligoelements, iodine, iron, zinc, selenium, copper, molybdenum, fluorine, manganese, boron, cobalt, and nickel are the most relevant ones. Iodine is very important as many people suffer from iodine deficiency. The bioavailability depends on the algae species and on the treatment during harvesting and processing [70]. According to this, it can be said that algae possess mineralogenic properties, which represents a useful tool, since these minerals are worthy in both fertilizer and animal feed [43].

Finally, fish farming, as well as intensive livestock production, produces effluents with a high content of inorganic nitrogen and phosphorus, compounds that are needed to produce a biomass of algae and have a high cost. Using these wastes for the production of algae biomass, environmental benefits could be obtained due to the seaweed biofiltration and depuration capacities, favoring the purification of effluents, as well as promoting a reduction of costs thanks to the use of waste waters as a source of necessary compounds [69].

## 5. Food and Technical Uses of Algae

Although algae extracts can be used in several products, they are mainly used as food ingredients in the formulation of food products (Table 4) [91,92]. In East Asia, as well as in the Pacific Islands, food has been based for centuries on the direct consumption of seaweed, the red seaweed Nori, or laver (*Porphyra*) being the most common commercial ones. In Japan, there are farms in shallow bays and seas comprising approximately 100,000 hectares. The *Porphyra* algae has a life cycle that includes two phases: A small and shell-boring. The first phase can be favored and augmented by humans, by planting on special platforms containing sea beds to which oyster shells are attached by using ropes or nets. In turn, the second phase consists on the germination of the conchospores and their growth along the platforms. It is at this time that the nets or ropes are removed to collect, wash and press the algae so that their drying is accelerated [93].

*Palmaria palmata,* also belonging to the group of red algae, is the most consumed in the north zone of the Atlantic Ocean. It is known by different names, such as Dulse, *duileasg*, *duileasc,* or *söl*, depending on the region, and its use as a food ingredient is quite widespread. In some areas of the mentioned regions it is also used as a flavor enhancer. Obtaining it is based on the hand collecting of the marine rocks in which they are trapped and accessible when the tide is low [93].

This method of manual collection is also carried out to obtain some brown algae, such as *Laminaria* and *Undaria*, in some Asian countries, such as Japan or Korea. [91], in which the food is supported robustly on the use of algae, using them for the accompaniment of numerous foods such as fish and meat, or for the elaboration of other recipes such as soups [94]. There are green algae whose leaves are similar to those of lettuce, belonging to the species *Monostroma* and *Ulva*, which are used precisely for the preparation of salads, although they are also included in soups and other types of dishes.

From a nutritional viewpoint, seaweeds are an alternative sources of proteins, certain brown species presenting much more protein content than other vegetarian sources, such as soy beans [95]. In the same way, their lipid content includes a concentration of fatty acids within 1 and 6 g/100 g of dry weight. In addition, it should be noted that some species have high concentrations of polyunsaturated fatty acids, namely eicosapentaenoic acid (up to 24%) [95].

**Figure 1:**
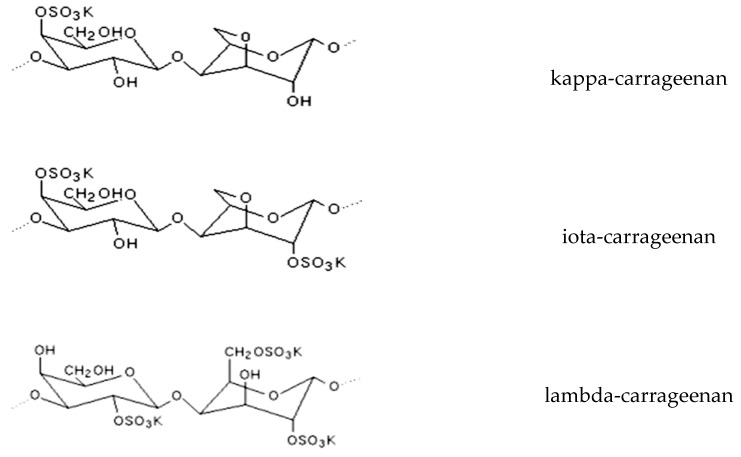
Different carrageenan types from red seaweeds.

One of the most important nutritionally relevant components of seaweeds are polysaccharides, most of them cannot be digested by humans, and thus, they can be considered as dietary fiber (33%–75% of the total composition) [96,97]. In turn, soluble fractions account between 50% and 85% of total dietary fiber content [96]. Some algae polysaccharides with particularly industrial relevance are described below:

Red algae (Rhodophyta) represent a particularly interesting group when considering polysaccharides, as they contain high quantity of sulfated galactans, such as agar or carrageenans. In the past decades, carrageenans have been used as natural ingredients in the elaboration of gels and as thickeners in a wide range of food applications [98]. They can be classified into three groups, according to their industrial use (kappa-, iota-, and lambda-), differing in the quantity and position of their sulfate ester substituents, as well as in the 3,6-anhydro-d-galactose content. The conformation of carrageenans is directly related with their technological properties (i.e., gelification, thickener). Kappa- and iota-carrageenans are gel forming and contain a 3,6-anhydro-galactose unit. In turn, lambda-carrageenans have only galactose residues and are used as thickeners. They lack of 3,6-anhydro-D-galactose ether linkages and, thus, the 4-linked substituent changes to a different conformation, disturbing their helical conformation [96] (Figure 1).

**Figure 2:**
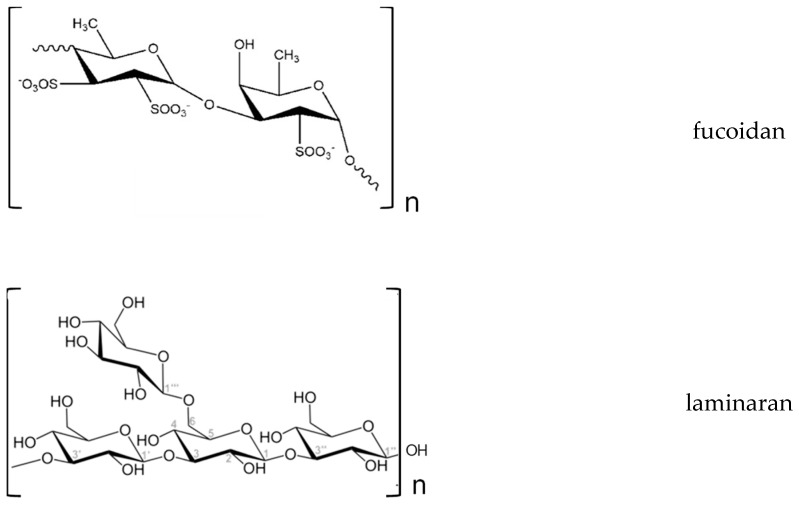
Structure of fucoidans and laminarans present in brown algae.

Fucans, another kind of polysaccharides, generally present in brown algae, are classified into three main groups: Fucoidans, glycorunogalactofucans, and xylofucoglycuronans. Fucoidans are the leading components of the soluble fiber present in such kind of algae [96]. They are branched polysaccharides sulfate esters, soluble in water and acid, consisting in (1→3) and (1→4)-linked-l-fucose residues, that may be organized as (1→3)-α-fucan chains or as alternating (1→3) and (1→4)-bonded α-l-fucose residues. The l-fucose residues are often substituted with sulfate (SO_3_) groups on C-2 or C-4 (rarely on C-3) [99,100]. Besides fucose, fucoidans also contain galactose, mannose, rhamnose, uronic acids, glucose, and xylose [99] (Figure 2).

Xylofucoglycuronans or ascophyllans consist of a polyuronide backbone, mainly poly-b-(1,4)-d-mannuronic acid branched with 3-*O*-d-xylosyl-l-fucose-4-sulfate or, sometimes, with uronic acid. Glycuronogalactofucans composition can be described as linear chains of (1,4)-d-galactose branched with l-fucosyl-3-sulfate or, sometimes, uronic acid at C-5 [101].

Laminarans are other type of polysaccharides present in Pheophytes (*Laminaria* species). They are responsible for the food reserve of brown algae. Although laminaran composition is species dependent, they contain of 20–25 units of glucose on average, and consist in (1,3)-β-d-glucan with β(1,6) branching [102]. Laminaran chains can be classified into two types (M or G): M chains have a mannitol residue at the reducing end, and G chains, a glucose residue [99] (Figure 2).

**Figure 3:**
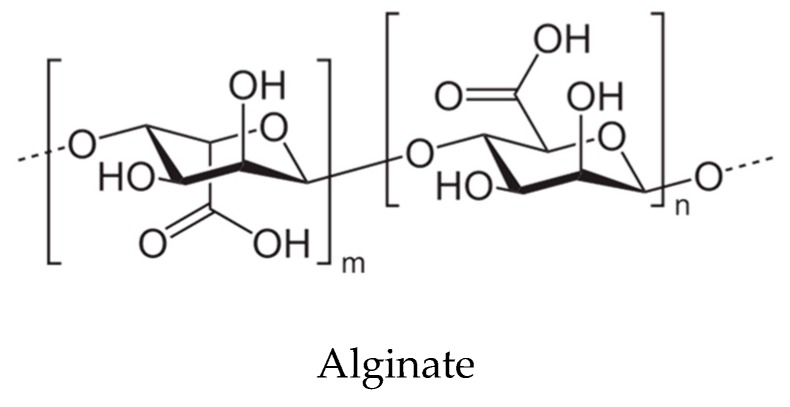
Structure of alginate.

Other remarkable compounds are alginic acid derivatives, also named alginates, that are constituted by linear polysaccharides with 1,4-linked β-d-mannuronic and α-l-guluronic acid residues distributed unregularly along the chain [99]. Alginates are present in brown seaweeds as sodium and calcium alginate, conforming highly viscous solutions, used for several technological applications (Figure 3).

The solubility in water of the above-mentioned algae polysaccharides certainly facilitates their commercial extraction. In fact, certain phycocolloids present in the cell walls of many algae can be easily extracted with hot water. Phycocolloids classification includes three major groups: Alginates, carrageenans, and agars, and are interesting because they are safe for humans and animals’ consumption. Many of these compounds are currently included in a wide range of processed and ready-to-eat foods, such as instant cakes or synthetic toppings [103]. An example is the use of industrially extracted alginates of brown algae species, such as *Macrocystis*, *Ascophyllum* and *Laminaria*, for the elaboration of ice cream, to avoid the formation of large amounts of ice and give them a creamy and smooth texture. Such alginates have been also used for the elaboration of energetic or sweet bars, and of dressings for salads as satiating agents, or in the preparation of syrups as thickeners and / or emulsifiers [104]. Alginates can also be extracted from red algae, such as *Gelidium*, *Gracilaria*, *Acanthopeltis*, *Pterocladia,* and *Ahnfeltia*, but their food use is reduced to special fillings for baking, canned food products, and as clarifiers in wine or beermaking. However, the use of agar in the R&D industry, as well as in microbiological and analytical laboratories, is really widespread, mainly for the preparation of culture media compatible with a wide variety of cell lines [105]. Carrageenans, also described previously, can be obtained from several species of red marine algae like *Eucheuma* (Philippines), *Chondrus* (United States), and *Iridaea* (Chile). Their main uses are thickening and stabilization of some dairy products like syrups, puddings or canned foods for animals, and they are also used in the elaboration of some medicines and some cosmetics as shampoos and creams [98].

During the 18th century, soda was obtained from brown macroalgae (*Phaeophyceae*) by collecting and scorching. In the following century, the main source of this compound was moved to the soda-rich mines discovered in Austria, among others, so the use of algae for this purpose drastically decreased. However, as the century progressed, they regained importance as a source of salts and iodine, so their exploitation suffered a tremendous boom, until the discovery of iodides and cooking salt, which practically plunged them back into disuse. Later, by the time of the First World War they were revalued for the production of fertilizers, such as potash, and acetone, used for the manufacture of smokeless gunpowder [106].

As mentioned above, algae have been used throughout the world as fertilizers for centuries. It is one of the most widespread uses of algae, as farmers from all coastal areas have collected them since ancient times, both at the oceans and reefs where they were trapped during storms or tides. Once harvested, they were left to dry spread out on the ground, obtaining a dry raw material with a mineral content that can amount up to 50% of the weight, in addition to high amounts of organic nitrogen derivatives. Currently, algae-based fertilizers that are commercialized include a set of micronutrients and macronutrients that help the plants grow properly [107].

## 6. Prebiotics from Algae

As shown in the previous section, macroalgae are responsible for the production of a large range of primary and secondary metabolites that can be used for different applications in food, cosmetic, pharmaceutical, and other industries [108]. Many of these compounds have well-established antiinflammatory, antimutagenic, antitumor, antidiabetic, hepatoprotective, free radical scavengers, anticoagulants, thrombolytic, and antihyperthensive properties [108,109]. In this section, the properties of non-digestible carbohydrates from algae will be addressed. A brief summary of those health beneficial effects is displayed in Table 5.

According to the last accepted definition, prebiotics are substrates that are selectively used by host microorganisms conferring health benefits [121]. As the target of most prebiotic compounds is the gut, such ingredients must not be hydrolyzed in the upper part of the gastro-intestinal tract. This ensures their safe arrival to the colon, where they stimulate the growth and/or activity of one or a limited number of bacteria, thus positively modulating the intestinal microbiota [121]. Although prebiotic properties must be well-demonstrated both in vivo and in vitro [121], the capacity to safe arrive to the gut converts non-digestible ingredients in potential prebiotic compounds (or prebiotic candidates).

### 6.1. Chemistry and Obtaining of Prebiotic Compounds from Seaweeds

Some prebiotics (or prebiotic candidates) naturally occurring in seaweeds are saccharides presenting a degree of polymerization (DP) within 2 and 9 (sometimes within 8 and 20). Some researchers consider up to 25 sugar residues [122]. Such oligo/polysaccharides include galacto-oligosaccharides (GOS), agarose-derived oligosaccharides (AGAROS), xylo-oligosaccharides (XOS), neoagaro-oligosaccharides (NAOS), alginate-derived oligosaccharides (ALGOS), arabinoxylans, galactans and glucans [114]. Although not all of them fulfill all the criteria requested for a compound to be considered as prebiotic (i.e., be refracting to hydrolysis and absorption in the upper part of the gastro-intestinal tract, selective promotion of bifidobacteria and/or lactobacilli in the colon, beneficial effect of their fermentation products, stability towards technological processes when incorporated into food products) [121], none of them is degraded by enzymes in the first part of the gastro-intestinal tract. Therefore, they achieve at least one of the requested criteria and take part of the dietary fiber.

Prebiotic oligosaccharides from seaweeds can be obtained by the hydrolysis of naturally occurring polysaccharides. This process has encouraged the development of new extraction techniques, as algae polysaccharides have a wide variety of chemical bonds and conformations, including α or β bonds, *cis* or *trans* configurations, d or l, or *R* or *S* chiral centers. This issue is of great importance since certain bonds can be hydrolyzed with enzymes, whereas others require other techniques as no natural enzymes are able to hydrolyze them [120,123]. Therefore, it is necessary to look for other techniques to obtain oligosaccharides from polysaccharides. Some authors [122] summarized the characteristics of different techniques, as well as their suitability to be used for the hydrolysis of some polysaccharides. Hence, the main non-enzymatic hydrolytic techniques used at an industrial level are ultrasound (in the case of carrageenans, agarose and xylan), microwaves (for hydrolysis of exopolysaccharides obtained from *Porphyridium cruentum*), use of free radicals (for fucoidan and algae galactan), or the application of diluted acids (for example, phosphoric acid) [124,125,126,127,128,129,130].

In the case of acids, they can be used as hydrolytic agents only for neutral polysaccharides, namely fucoidans, carrageenans, or galactans. In addition, certain components can be lost during the process and most of the glycosidic bonds are not specifically hydrolyzed, thus giving rise to various low molecular weight derivatives. In spite of that, phosphoric acid is capable of effectively hydrolyzing polysaccharides of some algae species, such as *Chlorella vulgaris* and *Spirulina platensis* [128], mostly composed of uronic acids and giving rise to interesting oligosaccharides with potential prebiotic properties [122].

The use of free radicals is another effective technique for the hydrolysis of polysaccharides, since it does not affect the structure of the compounds when it is correctly performed [122]. Other authors have demonstrated that this technique can be applied for the hydrolysis of fucoidans in low molecular weight compounds (≈8 kDa), although then it is necessary to apply other procedures for the purification of the obtained products [129]. Additionally, the obtained products demonstrated a better anticoagulant capacity with respect to the original polysaccharide [129].

The physical technique of microwaves also gives rise to glucidic compounds of low molecular weight (≈12 kDa) from natural polysaccharides extracted from algae. The obtained products do not undergo structural changes and have a surprisingly enhanced immunomodulatory and anticancer capacities with regard to the original substrate. The higher solubility of the products (with lower molecular weight) was remarked as a possible explanation for such observations [131]. Therefore, the use of this technique offers several clear advantages: it is economical, easy to use, non-toxic and is green, since the energetic and time consumption are not environmentally harmful [132]. All these techniques can be optimized to guarantee the highest results [133].

### 6.2. Prebiotic Properties of Oligo and Polysaccharides from Seaweeds

**Figure 4:**
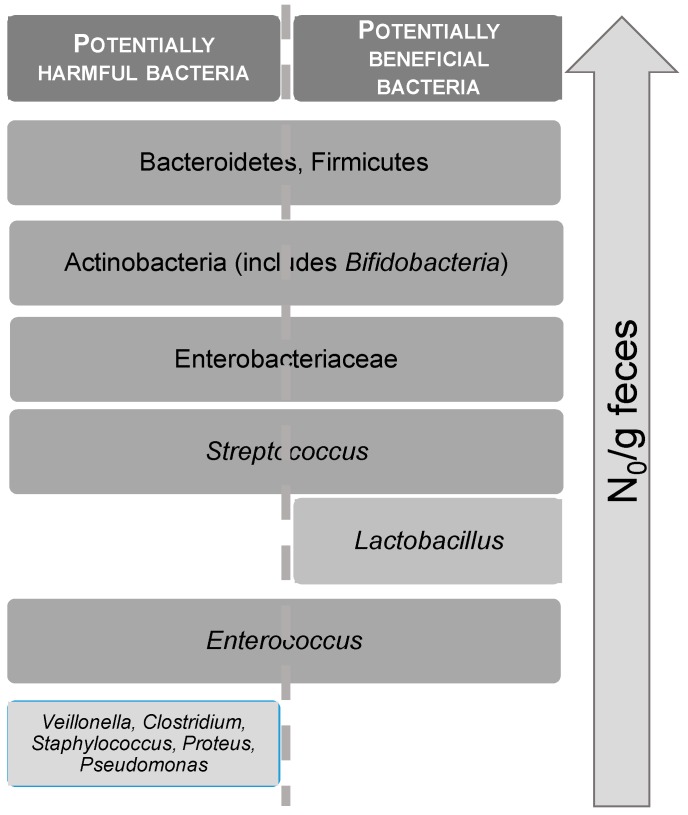
Beneficial bacteria and suppressing pathogenic microorganism.

Taking into account that some oligo and polysaccharides extracted from algae are not hydrolyzed in the upper part of the gastro-intestinal tract, they represent novel potentially useful raw materials for the obtaining of prebiotics [134]. To determine whether oligo and polysaccharides extracted from seaweeds are prebiotics, their fermentation by microorganisms from the intestinal microbiota is usually assessed [134]. First of all, the stability of algae polysaccharides when exposed to saliva, gastric and intestinal environments is assessed. The activity of intestinal microbiota is commonly evaluated by measuring metabolic end products, such as gases and short-chain fatty acids (i.e., acetic, propionic, butyric acids). Using molecular methods such as fluorescence in situ hybridization (FISH), polymerase chain reaction (PCR), denaturing gradient gel electrophoresis (DGGE) and 16S rRNA gene sequencing provides a complete representation of the in vitro effect of prebiotic candidates from seaweeds on the modulation of intestinal microbiota by increasing the amount of beneficial bacteria and decreasing prejudicial microorganisms [134,135] (Figure 4).

In turn, for in vivo studies, prebiotic candidates are orally administrated to the host (mouse, rats, monogastric, ruminants, and humans), and modifications in the composition of intestinal microbiota (Firmicutes/Bacteroidetes ratio, the two phyla present in the microbiota), and production of short chain fatty acids are determined. Recently, it was reported that polysaccharides obtained from the brown alga *Ascophyllum nodosum* increase the quantity of Bacteroidetes and Firmicutes, suggesting the potential of *Ascophyllum nodosum* polysaccharides to decrease the risk of obesity. Furthermore, the total short chain fatty acids content after fermentation increased significantly. These results suggest that *Ascophyllum nodosum* polysaccharides have potential uses as functional food components to improve human gut health [117]. Other authors evaluated the prebiotic properties of the brown seaweed *Ecklonia radiata* oligosaccharides in vivo, when administered the polysaccharide fraction of seaweed (rich in fucoidan and alginate) to rats. Such fractions lead to a decrease in the levels of toxic protein fermentation products, enhancement of the numbers of butyrate-producing *Faecalibacterium prausnitzii*, and decrease of the numbers of potentially pathogenic *Enterococcus*, thus demonstrating a potential prebiotic effect [136]. Some authors [137] demonstrated an increase in the population of bifidobacteria and lactobacilli both in the cecum and feces of rats fed with diets supplemented with alginate oligosaccharides. Such prebiotic effect was even greater than that of rats fed with a diet containing FOS, a well-established prebiotic. Similar increases were observed by [138] in mice fed with diets supplemented with agarose hydrolysates (NAOS). Moreover, a lower number of *Bacteroides* compared to the controls fed with FOS, were observed [135]. In addition, laminarin supplementation of rats feed also enhanced the cecal population of bifidobacteria, with no significant effect on lactobacilli. Moreover, laminarin also suppressed certain putrefactive compounds considered as risk markers for colon cancer, such as indole, cresol, and sulfide, and had immunomodulatory properties [139].

**Figure 5:**
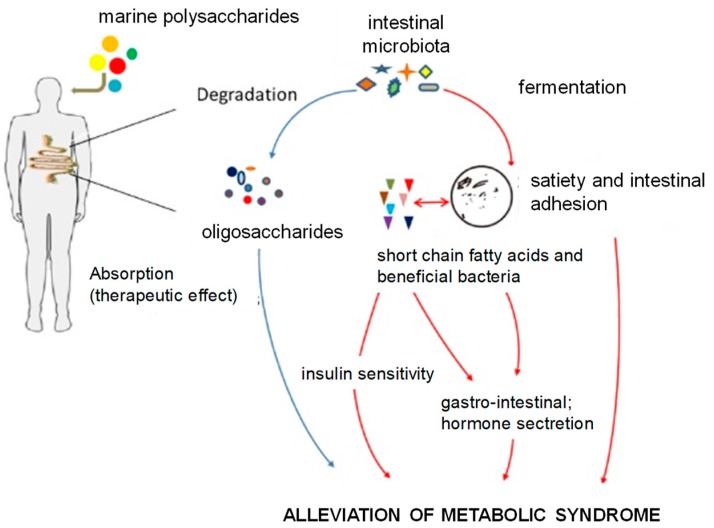
Effects of polysaccharides from marine algae on metabolic syndrome [116].

In turn, green algae, such as *Enteromorpha prolifera* and *Laminaria japonica*, spread all over the Chinese Qingdao coast, can also be fermented by intestinal microbiota [140]. A close relation between the metabolic products of polysaccharides from marine algae and the regulation of enteroendocrine hormone secretion, blood glucose, and lipid metabolism has been recently reported, thus suggesting their effect on alleviation of metabolic syndrome symptoms [116,141] (Figure 5).

The prebiotic effect of polysaccharides from seaweeds has been also evaluated on farm animals [135]. Many studies have evaluated the effects of seaweed polysaccharides (i.e., laminarin) in pigs, lambs, or cattle [142]. However, the differences in digestive physiology and anatomy must be considered when attempting to extrapolate data from ruminants (cattle and sheep) to monogastrics, such as pigs. Some in vitro studies in some animals such as pigs, rabbits, birds, and some ruminants conclude that some algae have the ability to meet the energy and protein requirements for healthy growth, while other algae contain certain compounds that have biological activities, so that both types could be used as prebiotics to favor the breeding of such animals [93].

To summarize this section, algae are an excellent natural source of natural polysaccharides, that can be extracted and hydrolyzed for the obtaining of prebiotic saccharides. Extraction processes based on physical methods are the most efficient ones, since they present minimal or no adverse effects on human health. Besides that, these methods do not alter the original structure of the compounds and are environmentally friendly [132]. Additionally, many of the compounds obtained show good bioactive capacities, so their use as prebiotics is highly recommended [143].

Regarding the functionality of seaweeds, further studies are still necessary to gain information about their intestinal benefits, and if mixtures of polysaccharides can be tailored to improve health benefits. This would be of great importance for the formulation of functional ingredients, improving fermentability by gut microorganisms. In this context, it appears that in the future, human beings could modulate the microbiome through the consumption of drugs and prebiotics, which could be at the cutting edge in the prevention of some diseases [116].

## 7. Antioxidants from Algae

In spite of having previously named the properties of antioxidants in algae, it is necessary to expand the given information because there are many different compounds, of different natures, that present this quality, whether they are water-soluble (i.e., phenolic compounds or vitamins), or soluble in apolar solvents (i.e., carotenoids or tocopherols) [143].

### 7.1. Carotenoid Pigments

Carotenoids are one of the most known and widespread natural pigments in the world. Their structure corresponds to that of hydrophobic tetraterpenes with a structure of branched hydrocarbons with a methyl group bound at C-40 [144,145]. The pigmentation produced by carotenoids has to do with the conjugated double bonds that appear in the carbonate skeleton, since they have the ability to capture photons at different wavelengths of the visible spectrum. All organisms that carry out photosynthesis contain carotenoids, which does not mean that they are exclusive compounds of these autotrophic organisms, since they are also present in some bacteria and fungi [146]. More than 600 types of carotenoids are known, which can be divided into two large groups according to their molecular structure: Carotenoids and xanthophylls. The first group is characterized by having linear or cyclic hydrocarbons at one or both ends of the molecule (like β-carotene). In turn, xanthophylls are described as oxygenated derivatives of carotenes (such as violaxanthin), and can be found in green algae, as well as in more developed plants, although there are compounds (i.e., canthaxanthin and loroxanthin) that are exclusive from green algae [147,148]. Similarly, brown algae and some flagellated microalgae are capable of producing another type of xanthophylls, such as diatoxanthin or fucoxanthin [149].

Carotenoid essentially plays a photoprotector role in algae, preventing their photosynthetic system from damage. In addition, they also participate in processes such as phototaxis (movements resulting from the intensity of light) or phototropism (movements in the direction of the light). Some microalgae are capable of generating high amounts of carotenes in response to external stimuli that cause stress, so that they can adapt to new changes [150]. The microalgae species producing the highest amounts of carotenoids are *Dunaliella salina*, which produces β-carotene, and *Haematococcus pluvialis*, which produces astaxanthin. Currently, the use of β-carotene is widespread in the industry because it is a natural ingredient that provides color to matrices, either in food (such as cheeses and butter) or cosmetic formulations [151]. In addition, carotenoids have an associated provitamin A activity. Despite being less known, astaxanthin is also a skin and eye protector, muscle strengthener, immune modulator, and coloring agent. There are studies claiming that the daily ingestion of this astaxanthin slows down the aging process, as it has a regenerative capacity and is a free radical scavenger [152].

### 7.2. Phycobilin Pigments

Phycobiliproteins are secondary pigments generated by microalgae, which help them to exploit the light energy while protecting them from harmful radiation. It seems that the antioxidant mechanisms within the organisms that create them are very similar to those carried out in food matrices or in the human organism [153]. The main microalgae producing these compounds are cyanobacteria (*Spirulina*, which produces phycocyanin, blue) and cryptomonads, but are also found in red algae (*Porphyridium*, which produces phycoerythrin, red). Despite being a labile molecule, phycocyanin is capable of generating a blue color that other natural dyes do not achieve, so it is used in the manufacture of foods such as ice cream, yogurt, chewing gum, and beverages, as well as in various cosmetic products, mainly in Japan [154].

### 7.3. Phenolic Compounds

Phenolic compounds are a group of secondary metabolites comprising a wide variety of compounds produced by both terrestrial and aquatic plants, which include, of course, algae [155]. Despite the well-known diversity of structures of phenolic compounds, they must possess a benzene ring having at least one hydrogen substituted with a hydroxyl group. One of their most outstanding features is their antioxidant properties, as they prevent the formation of many free radicals because of their metal ion chelating capacity [156,157]. Phenolic compounds are commonly classified into 5 large groups: Flavonoids (the largest subgroup and associated with different bioactivities, among which, as previously described, the antioxidant, and radical scavenging activity) [158,159], lignans, tannins, tocopherols, and phenolic acids [160]. They are common compounds in algae, especially in brown ones, since some species of brown seaweed have phlorotannins, which are polymers of phloroglucinols (1,3,5-trihydroxybenzene) that can reach up to 15% of the dry weight of these algae [161]. In addition, they are composed of up to eight rings interconnected with each other, which give antioxidant properties much higher than many polyphenols obtained from terrestrial plants, since most of them contain only three or four rings in its structure [155].

### 7.4. Vitamins and Minerals

Vitamins are well-known for their high antioxidant capacity. However, when attempting to reproduce their synthesis artificially, a decrease in activity with respect to those obtained directly from natural matrices was observed [162]. That is why new natural sources containing essential vitamins are continuously searched. Among them, microalgae biomass contains most of the essential vitamins (e.g., A, B1, B2, B6, B12, C, E, nicotinate, biotin folic acid, and pantothenic acid), as well as an interesting variety of minerals (e.g., Na, K, Ca, Mg, Fe, and Zn) [163]. In fact, some species of microalgae contain high levels of some essential vitamins that are requested in higher quantities by humans. This is the case of B12 vitamin, which can be combined with a mineral content, such as iron, thus converting it in an ideal dietary supplement for vegetarian diets. An example of such combination is Spirulina. However, and as expected, not all algae contain the same amounts of the same vitamins. The vitamin content of each species of algae depends on endogenous factors, such as their life cycles, their growth status and genotype, as well as on exogenous factors, such as the amount of nutrients in their habitat, the intensity of the light reaching them and the processes they suffer since they are collected. Therefore, there are several different factors that can be modulated to achieve the desired amounts of algae vitamins [33].

## 8. Production and Consumption Statistics and Future Markets

**Figure 6:**
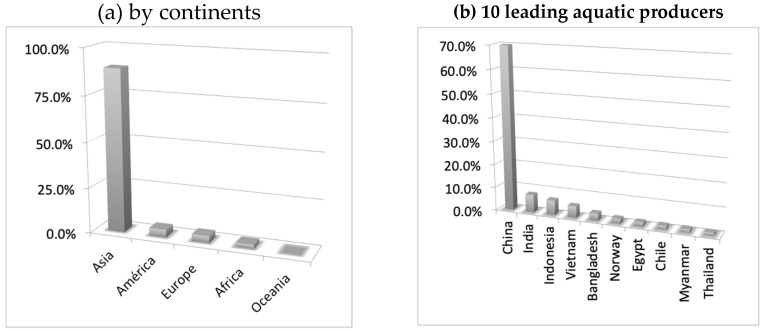
(**a**) Distribution by continents of world aquiculture production (2017) (Source: FAO [31,164,165,166]) and (**b**) production of the 10 leading aquatic producers in the world (2017) (Source: FAO [31,164,165,166]).

**Figure 7:**
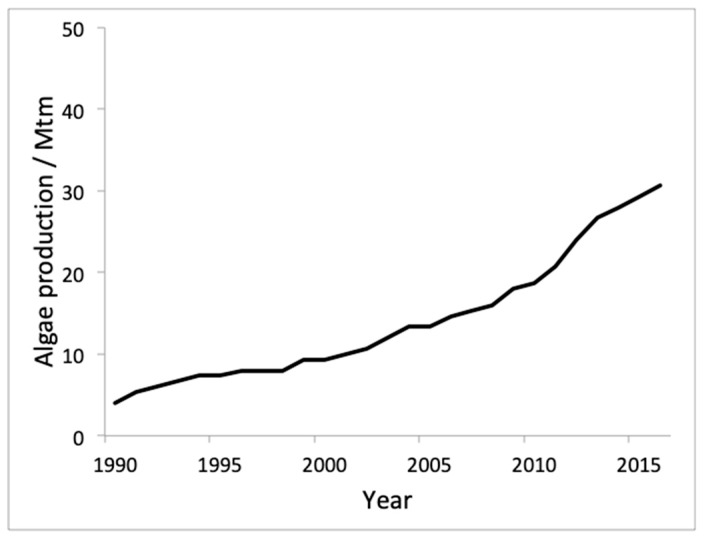
Time evolution of world aquaculture algae production in millions of tons from 1990 to 2016 (source: FAO [31,164,165,166]).

A senior FAO official [31,164] announced in 2017 during the High Level International Meeting on the Global Initiative “Blue Growth” for Latin America and the Caribbean, held in Mexico City, that “aquaculture worldwide is the productive sector with the greatest growth, exceeding as of 2011 the growth rates of bovine cattle”. Thus, according to statistics from 2015, the world aquaculture production consisted in 51.9 Mt of fish (68%), 16.4 Mt of mollusks (21%), 7.4 Mt of crustaceans (10%), and 0.9 Mt of other aquatic animal species (1%). In particular, aquaculture in inland waters represents the most important sector of the production of edible organisms (43.6 Mt), which represents 59% of world production. The average annual growth rate of aquaculture production for 2001–2015 was 5.9%, significantly lower than in the last two decades of the 20^th^ century, which stood at 10.8% and 9.5%, respectively. However, the contribution of aquaculture to production has been increasing steadily, reaching 45% in 2015 from 26% in 2000. If we focus on the world production of aquatic plants, mainly marine algae, reached 30.5 Mt in 2015. In this case the extractive processes are merely testimonial since 96% of the production (29.4 Mt) were obtained through aquaculture. Analyzing the distribution of aquatic production at the continental level and according to statistics for 2015, it would be headed by the Asian continent with a production of 68.4 Mtm. America and Europe would be at a distance with 3.3 Mtm and 3.0 Mtm. Africa produced the order of 1.8 and Oceania would scarcely contribute with 0.2 Mtm. The percentage of world production is shown in Figure 6. Figure 7 shows the weight of the top 10 world producers in aquaculture. Table 6 presents the evolution in Mtm of the production of the main producers in aquaculture in recent years.

Access to statistics on seaweed production is largely dependent on annual publications of the Food and Agriculture Organization of the United Nations (FAO), such as ‘The State of World’s Fisheries and Aquaculture.’ The report published in 2018 only comprises production statistics for 2016 [164]. Table 6 shows the production in aquaculture (Mtm) of main producers in 2015–2016.

Meanwhile Figure 6 shows the distribution by continents of world aquiculture production (2017) (Source: FAO) and the production of the 10 leading aquatic producers in the world [31,164,165,166].

In 2016, aquaculture was the source of 96.5% of the total volume of 31.2 million tons, including wild-collected and farmed plants together. When it comes to aquatic plants cultivation, it mainly refers to seaweeds. Their cultivation almost triplicated in the last ten years. This fact supports the increase in the use of algae, although the demand still exceeds the supply offered by aquaculture. Conversely, the production of algae is decreasing (~5%) because of the collection of natural habitats [164]. Figure 7 shows the world evolution of cultivated aquatic plants.

Macroalgae have a large number of applications being the most relevant the production of human food products [74] and 15% is attributed to algae extracts, like hydrocolloids for their use in fertilizers, animal feeds and for its biological activities [167]. Nowadays, 221 species of algae are collected globally, from which 145 are for human nutrition and 101 for phycocolloid production. These include 125 rhodophytes, 64 phaeophytes and 32 chlorophytes [168]. From these, ten are largely cultivated, specially the brown ones *Laminaria japonica, Undaria pinnatifida* and the red ones, *Porphyra* spp., *Porphyra tenera, Eucheuma* spp., *Kappaphycus alvarezii* and *Gracilaria* spp. and *Gracilaria verrucosa* [169].

In 2010, 19 million tons of aquatic plants were produced in aquaculture globally, being sewed the largest percentage by far with 99.5% of the total. Of all the seaweed used in the world in 2010, 99.5% was prevenient from aquaculture, being a growing economy with a 7.7% per year increase since the 1970s [170]. The production of Algae is dominated by countries in East and Southeast Asia, reaching to 99.6% of quantity in 2010. China has the major portion of the production, contributing with approximately 58% of worldwide cultivated algae production by quantity. Indonesia, Philippines, South Korea, Japan, and North Korea are some of the other major seaweed producers. Japan has a high value of production, ranking in third position, due to its high-value of *Phorfira* production, accounting for 20% [165,166]. Almost all cultured species produced in East Asia are used for human consumption, with the exception of a small amount of Japanese brown algae that is also used as a source for the extraction of algin and iodine. Contrary, in Southeast Asia, *Eucheuma* algae are the principal cultivated species, used principally as a source for carrageenan obtaining. Aside Asia, a small fraction is also cultivated in Zanzibar (Tanzania) and Chile.

Nowadays, algae have several important uses in the food, cosmetic, and fertilizers industries and in the production of hydrocolloids, such as alginates and agar, being the last one the mainly current commercial exploitation. Furthermore, the production of bioactives and future commercial opportunities for seaweed compounds has increased and more than 15 000 different molecules have been isolated to date [170] hoping to be incorporated in human food, nutraceuticals, pharmaceuticals and animal stock feeds—but also fertilizers, biofuels, and soil conditioners. Opportunities for utilization aquafeeds, in food, feed, and nutraceuticals have presented as offering the most promising results in a reasonable period [171]. The production of seaweed globally has increased over the years as well has consequently the by-products and co-products derived from them.

## 9. Conclusions on Trends and Challenges for the Sector

The term “functional food” can be defined as foods claiming to have additional function(s) by incorporating new ingredients or greater amounts of the existing ones. Such ingredients, called “functional ingredients” have specific health benefits, including immunomodulation, and reduction of the risk of certain diseases, among others. Seaweeds that can be consumed by humans appear as innovative resources with an interesting potential to be used as functional ingredients. Some of them contain high insoluble dietary fiber. In particular, the high content of non-digestible polysaccharides converts marine algae in a rich source of prebiotics or prebiotic candidates. Further investigations about prebiotic properties both in vitro and in vivo are still necessary to incorporate such ingredients in the formulation of functional foods. On the other hand, certain secondary metabolites from marine algae, including antioxidants, are able to decelerate or prevent oxidation processes, thus being favorable health related compounds. Therefore, there is a need to solve problems such as the determination of the identity of the harvests as fresh materials for functional foods, and the regularization of the products, because seaweeds from different locations can produce different levels of active compounds.

In addition of being a source of novel, beneficial, and natural products, algae and their cultivation offer advantages on a larger scale, since aquaculture is an environmentally friendly process and favors the production of compounds rich in biomass and proteins that could supply or mitigate industries with many more toxic waste. Furthermore, the implementation of this technique in developing countries could entail great financial advantages. However, to do so, it would be first necessary to solve problems, such as the lack of marine spaces, where to carry it out, or the initial economic support. For the development of the aquatic plants cultivation industry (i.e., algae), the transfer of technological improvements (i.e., creation of sea beds not requiring coastal areas) to tropical areas would be very convenient. This way, the problems of lack of cultivable surfaces would be overcome.

In conclusion, governments shRedondelaould become part of this booming industry, supporting sustainable development through investments in infrastructure, personnel, materials, and projects aimed at research, development and innovation, thus committing to a green future for our planet.

## Figures and Tables

**Table 1:** Phylogenetic summary of the most common macroalgae.

Kingdom	Phylum/Division	Classes	Orders
Chromist	Ochrophyta	Phaeophyceae	Ascoseirales; Desmarestiales; Discosporangiales; Dictyotales Ectocarpales; Fucales; Laminariales; Nemodermatales Ralfsiales.
Plantae	Charophyta	Charophyceae; Chlorokybophyceae; Coleochaetophyceae; Klebsormidiophyceae; Mesostigmatophyceae; Zygnematophyceae.	
	Chlorophyta	Ulvophyceae	Bryopsidales; Cladophorales; Dasycladales; Oltmannsiellopsidaes; Trentepohliales; Ulotrichales; Ulvales
	Rhodophyta	Bangiophyceae; Compsopogonophyceae; Florideophyceae; Porphyridiophyceae; Rhodellophyceae; Stylonematophyceae.	

**Table 2:** Pigment content of the 3 common groups of macroalgae. Bold pigments represent the predominant ones in each group.

Pigment Class	Green Algae	Brown Algae	Red Algae	Reference
Chlorophylls	Chlorophyll *a* and *b*, and derivatives	Chlorophylls *b* and *c*, and derivatives	Chlorophylls *a* and *d,* and derivatives	[16]
Carotenoids	β-carotene, xanthophylls	Fucoxanthin and xanthophylls, β-carotene	Xanthophylls	[13,16,17]
Phycobiliproteins	-	-	Phycoerythrin and phycocyanin	[13,16]
Example	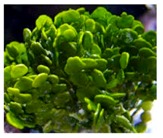 *Halimeda sp*	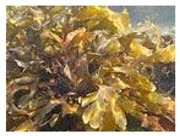 *Fucus serratus*	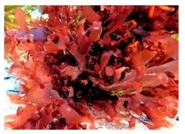 *Palmaria palmata*	

**Table 3:** Current marine algae applications.

Applications	Specific	Authors
Anti-biofilm activity		[39,40,41]
Biofuels		[32,42,43,44,45]
Bioremediation		[46,47,48]
Contraceptive activity		[49,50]
Cosmeceuticals		[51,52]
Fertilizer		[32,43,53]
Fish feed		[32,43]
Food ingredients		[32,43]
Pharmacology/medical	General	[32,54]
	Antibiotic, antiviral, antifungal activity	[55,56,57,58]
	Anticancer	[56,59,60]
	Anticoagulant	[61,62,63,64]
	Anti-inflammatory	[65,66,67]
	Antioxidants	[56,68]
Other applications:	Filter	[69]
	Mineralogenic	[43,70]

**Table 4:** Food and feed applications of the main red, brown and green macroalgae.

Name	Applications	Region/Country	References
Red Alga (Rhodophyta)			
*Porphyra* (alga nori)	Cultivated for food	Asia	[93]
*Saccharina japonica*	Cultivated for food	Japan	[93]
*Palmaria palmata* (Dulse)	Culinary ingredient, flavor-enhancer	USA, Canada, Scotland, Ireland, Iceland	[93]
*Gelidium* sp.*, Gracilaria* sp.*, Pterocladia* sp., *Acanthopeltis* sp.*, Ahnfeltia* sp.	Instant pie fillings, canned meats or fish, bakery icings, beer and wine clarifiers	Asia	[93]
*Eucheuma* sp.*, Chondria* sp.*, Iridaea* sp.	Thickening and stabilizers, imitation of creams, puddings, syrups, canned pet foods.	Philippines, Ireland, Chile, USA, Canada	[93]
*Grateloupia* sp.	As vegetable	Indo-pacific region	[94]
Brown Alga (Pheophyta)			
*Sargassum fusiforme,* *Sargassum dentifolium*	Farmed in small quantities (poultry, improves quality of eggs)	Europe, Asia, North America	[93]
*Ascophyllum nodosum*	Animal feed (ruminant and poultry diets), human consumption	Norway, UK, Portugal, USA	[93]
*Undaria* sp.*, Hizikia* sp.	Fried in oil, boiled in soup	Japan, Korea, China	[91]
*Macrocystis* sp.*, Laminaria* sp.	Ice-creams, syrups, salad dressings (texturizers, emulsifiers, thickeners)	Europe, USA	[93]
*Ascophyllum* sp.	Land animal feed (i.e., ruminants)	Iceland	[93]
*Laminaria digitata, L. hyperborea, L. latissima*	Animal feed	Europe, Asia	[91]
*Laminaria japonica*	Soup, fried in oil, with soy sauce	Asia	[91]
*Fucus vesiculosum*	Pigs diet	Sweden	[93]
*Enteromorpha prolifera*	Poultry diet	Europe	[93]
*Pelvetia canaliculata*	Pigs diet, human consumption during times of famine	Scotland, Ireland	[93]
Green Alga (Chlorophyta)			
*Caulerpa* sp.	Farmed in small quantities (poultry, improves quality of eggs), food (“green caviar”)	Europe, Asia, Northamerica	[93]
*Monostroma* sp.	Salads, soups, relishes, meat and fish dishes	Europe, Asia	[93]
*Ulva lactuca*	Lambs feed, soups, salads	Europe, USA, Asia, Australia, New Zealand	[93]
*Ulva intestinalis*	Rabbits feed	Egypt, Saudi Arabia	[93]
*Chaetomorpha linum*	Lambs’ feed	Tunisia	[93]

**Table 5:** Prebiotic or prebiotic candidates extracted from marine algae.

Prebiotic/Prebiotic Canditate	Origin/Source	Health Beneficial Effects	References
AGAROS (agaro-oligosaccharides)	Pheophyta (brown algae)	Immunomodulatory (decrease of pro-inflamatory cytokynes) antiinflammatory, carcinostatic, antioxidant, hepatoprotective, and α-glucosidase inhibitory activities	[110]
NAOS (neoagaro-oligosaccharides)	*Gracilaria* sp., *Monostroma* sp.	ROS scavenging, antioxidant and immunomodulatory effects, stimulation of lactobacilli and bifidobacteria populations	[110,111,112,113]
COS (carrageenan-oligosaccharides)	*Kappaphycus* sp., *Porphyria* sp., *Gracilaria* sp.	Immunomodulation, skin whitening, and moisturizing, stimulation of lactobacilli and bifidobacteria populations, repair of intestinal damage	[110,113,114,115]
ALGOS (alginate-oligosaccharides)	*Ascophyllum* sp.*, Fucus* sp.*, Undaria* sp., *Sargassum* sp., *Laminaria* sp. and *Macrocystis* sp.	Reactive oxygen species (ROS) scavenging, antioxidant and immunomodulatory effects, weight control, reduction of cholesterol, diabetes control (hypoglycemic and hypolipidemic properties), promotion of fecal microbiota metabolism, production of short chain fatty acids by the gut microbiota; decrease of putrefactive compounds and microorganisms, decrease of metabolic syndrome risk	[115,116]
Fucoidans (FUCOS)	*Cladosiphon* (aka Okinawa) *Ascophyllum* (*nodosum*), *Fucus* sp. *Sargassum* sp.	Hypocholesterolaemic, immunomodulatory, anti-obesity, anti-hyperlipidemia, attenuation of hepatic steatosis, anti-diabetes (reduction of insulin resistance), anti-hypertensive, antioxidant	[99,114,116,117]
	*Fucus evanescens*	Anticoagulant, antioxidant	[109,118]
	*Fucus vesciculosus*	Anticancer, antimetastatic	[109]
Galactofucans	*Laminaria japonica*, *Sargassum* sp.	Anti-lipidaemic, increases HDL, antiviral, antitumor, immunomodulator, antioxidant, neuroprotective	[114]
	*Undaria pinnatifida*	Antiviral, anticoagulant, antitumor, anti-proliferative, immunomodulatory, anti-inflammatory induced osteoblastic differentiation	[119]
	*Dictyota menstrualis*	Peripheral anti-nociceptive, anti-inflammatory, antioxidant; anticoagulant, anti-proliferative	[119]
	*Lobophora variegata*	Antioxidant, anticoagulant, anti-inflammatory	[119]
	*Adenocystis utricularis*	Antiviral	[119]
Xylo-galactofucans	*Spatoglossum schröederi*	Anti-thrombotic; Peripheral anti-nociceptive; Anti-proliferative, anti-adhesive, antioxidant	[119]
Arabinoxylans	*Ascophyllum*	Modulation of intestinal microbiota	[120]
Glucans	*Chlorela vulgaris*	Antitumor, infection preventive agent	[119]
Laminarin	*Ascophyllum* sp., *Fucus* sp., *Laminaria* sp., *Saccharina* sp., *Undaria*, *Enteromorpha* sp.	Antilipidemic, hypocholesterolaemic, fast decrease of blood glucose	[114,116]

**Table 6:** Production in aquaculture (Mtm) of main producers in 2015–2016 (Food and Agriculture Organization of the United Nations [31,164]).

Country	Production/Mtm	
	2016	2015
China	47.6	41.1
India	5.2	4.2
Indonesia	4.3	3.1
Vietnam	3.4	3.1
Bangladesh	2.1	1.7
Norway	1.4	1.3
Egypt	1.2	1
Chile	1.1	1.1
Myanmar	1	0.9
Thailand	0.9	1.2
